# Comparing the performance of narrow vs. broad search strategies when using machine learning-based software for title/abstract screening

**DOI:** 10.5195/jmla.2026.2286

**Published:** 2026-04-01

**Authors:** Michelle Swab

**Affiliations:** 1 mswab@mun.ca, Public Services Librarian, Health Sciences Library, Memorial University of Newfoundland, St. John's, NL, Canada

**Keywords:** AI, machine learning, evidence synthesis as topic, systematic review as topic, Screening Tools, search strategy development

## Abstract

**Objectives:**

To retrospectively evaluate workload implications and recall performance of narrower or broader database search strategies when using active learning screening tools.

**Method:**

A convenience sample of 10 completed reviews was used to assess search strategy performance in ASReview LAB, an open-source systematic review software tool. For each review, a single database search strategy was selected and then revised to either broaden (n = 9) or narrow (n = 1) the scope. Results from both the more sensitive (broader) and more precise (narrower) search strategies were labeled as relevant or irrelevant based on inclusion in the completed review. The labeled result sets were uploaded into the ASReview LAB simulation module, which mimics the process of human screening. Metrics such as number of records screened to reach recall of 95% or more were recorded. The effects of three different stopping rules on workload and recall were also explored.

**Results:**

For quantitative systematic reviews, the difference in absolute screening time required to reach 95% recall between broader or narrower search strategies was minimal (≤35 minutes). In contrast, for qualitative systematic reviews and other review types, broader search strategies led to increased workload. With respect to stopping rules, the time-based stopping heuristic resulted in substantial workload increases when broader search strategies were employed.

**Conclusion:**

Time savings achieved through the use of semi-automated screening tools may not always offset additional screening time required by broader, more sensitive search strategies. Librarians and information specialists should consider a variety of factors when determining the appropriate balance between search sensitivity and specificity in the context of semi-automated screening tools.

## INTRODUCTION

Evidence synthesis projects can be resource intensive, with many reviewers considering title and abstract screening to be particularly time-consuming [1,2]. Because of the substantial workload and perceived tediousness of manual title and abstract screening, many articles included in recent scoping reviews on evidence synthesis automation focus on this step [3,4].

A variety of automation tools and methods have been developed to reduce title and abstract screening burden. Some tools automatically identify certain study designs or types of studies, such as randomized controlled trials [5]. Others employ active learning algorithms that iteratively “learn” from reviewers’ inclusion and exclusion decisions, using this feedback to reorder the records presented for screening [6]. This process of re-prioritization can improve efficiency by displaying the most relevant abstracts earlier in the screening process. Re-prioritization may occur continuously or after a predefined number of records have been reviewed.

In some systematic review software tools such as Covidence, records are re-prioritized through active learning algorithms; however, screening remains fully supervised by human reviewers [7]. This means that human reviewers continue to assess each abstract and make inclusion and exclusion decisions. In other software, stopping rules may be applied to automatically exclude a proportion of the reprioritized records without human review. This kind of screening process is frequently described in the literature as “semi-automated” [8]. Estimates of workload savings achieved through active learning prioritization software and automated exclusions vary widely, ranging from 33-90% in one scoping review [9].

As of July 2025, active learning screening tools that automatically eliminate records are not recommended for Cochrane reviews. In the Cochrane Handbook, Lefebvre et al. note that “more work is needed to develop and validate safe stopping rules” so that no relevant studies are automatically excluded [10]. Additional barriers to adoption of automation tools in systematic reviews identified in the literature include lack of trust and setup challenges [11]. Nevertheless, active learning screening tools likely will achieve greater acceptance and uptake over time. Cochrane Handbook authors observe that stopping rule challenges are “not insurmountable” [10], and a new joint Methods Group between Cochrane, the Campbell Collaboration, JBI and the Collaboration for Environmental Evidence (CEE) is working to develop policies and guidance about the responsible use of AI in evidence synthesis studies. Additionally, the Guidelines International Network has recently published a set of principles for the use of AI in guideline development [12]. Lastly, a recent guidance paper on automation software in rapid reviews suggests that automatic exclusion of records may be acceptable for this review type [13].

Given the increasing acceptance and adoption of active learning screening tools in evidence synthesis projects, it is important for librarians and information specialists to understand the impact of database search sensitivity and specificity on title and abstract screening workload in semi-automated reviews. Search sensitivity (or recall) refers to the proportion of relevant articles retrieved as a percentage of all relevant articles; search specificity (or precision) refers to the proportion of relevant articles as a percentage of the number of articles retrieved [14]. While search sensitivity is traditionally prioritized in systematic reviews, the need for high recall tends to be balanced against the resources of the review team [15,16].

Developers of active learning prioritization tools argue that automating exclusions reduces screening workload, thereby allowing for broader database searches and minimizing the likelihood of missing relevant records [17]. An increased emphasis on broader database searches and improved search sensitivity may also help alleviate some of the burden on information specialists, particularly when addressing complex topics where achieving an optimal balance between recall and precision can be challenging and time-consuming. On the extreme end of the record retrieval spectrum, a preliminary study has assessed text mining prioritization of impractically large searches (>800,000 records) for two public health scoping reviews [18].

To date, there have been no published empirical studies about search strategy formulation in the context of active learning screening tools. One guidance article recommends performing the highest quality search strategy, “regardless of yield” [19]. However, this recommendation is not based on empirical evidence and does not address the complexities of balancing search recall and precision. This study explores how narrower or broader searches influence screening workload and recall when semi-automated active learning screening tools are used.

## METHODS

A retrospective simulation study was performed in ASReview LAB [20] using a convenience sample of 10 previously conducted reviews. A convenience sample refers to a set of cases selected based on accessibility and availability rather than through random sampling. I collaborated on these reviews and received permission from all principal investigators to use the review data for this project.

ASReview LAB is a free, open-source machine learning tool developed by researchers at Utrecht University. Like other active learning screening tools, ASReview LAB prioritizes records based on a reviewer’s previous inclusion and exclusion decisions, enabling faster identification of likely included studies. No set stopping rule is applied by the software itself; review teams must decide when screening will be discontinued. ASReview LAB features a simulation mode, which was used for this study. Simulation mode allows researchers to estimate the potential time savings associated with various datasets and different active learning models.

The sample reviews varied in their methods, and included systematic reviews of quantitative studies, systematic reviews of qualitative studies, scoping reviews, and an integrative review (see Table 1 for review characteristics). For each review, I selected a single database search strategy based on which database was expected to retrieve the greatest number of articles included in the original systematic review, as well as the availability of database features. For example, Ovid Medline provides opportunities to adjust adjacency operators to broaden searches. I also considered database variety in my database selection decisions. The database search strategy was then revised to either broaden (n = 9) or narrow (n = 1) the scope of the search. Decisions regarding search modifications were made using a real-world approach; I made changes to the search strategies that I may have made if the team had more or fewer resources at the original time of screening. Original and revised search strategies can be found in Appendix A. Both the original and revised searches were run in the selected database, with both searches limited to the original search date.

**Table 1:** Review project characteristics. Because these reviews used a multi-pronged search strategy and included multiple databases, it was possible for the expanded database search strategy to retrieve more, or fewer, included articles than the original search.

Review Project	Review Type	# of articles included in the completed review	Database	Search change	Original search retrieval for selected database (# of articles)	Included articles retrieved by original database search	Search strategy recall, original database search (%)	Search strategy precision, original database search (%)	Revised search retrieval for selected database (# of articles)	Included articles retrieved by revised database search	Search strategy recall, revised database search (%)	Search strategy precision, revised database search (%)
SGLT2 safety [21]	Systematic Review (Quantitative)	109	PubMed	Replaced precision maximizing with sensitivity maximizing methods filter	771	105	96.3%	13.6%	1709	105	96.3%	6.1%
Chronic pain peer support [22]	Systematic Review (Quantitative)	24	Embase	Changed major headings to regular headings	1421	23	95.8%	1.6%	3909	23	95.8%	0.6%
Cannabis purchase choice [23]	Systematic Review (Quantitative)	35	Ovid Medline	Removed concept group relating to purchase choice	2674	16	45.7%	0.6%	3311	16	45.7%	0.5%
Production effect [24]	Systematic Review (Quantitative)	26	APA PsycInfo	Removed concept group relating to methods	469	16	61.5%	3.4%	1557	16	61.5%	1.0%
Autism diagnosis experiences [25]	Systematic Review (Qualitative)	36	CINAHL	Removed concept group relating to methods	1248	16	44.4%	1.3%	3730	16	44.4%	0.4%
Chronic illness & workplace policy [26]	Systematic Review (Qualitative)	44	CINAHL	Removed concept group relating to methods	735	24	54.5%	3.3%	3879	24	54.5%	0.6%
ICU sustainability [27]	Scoping Review	99	Ovid Medline	Replaced adjacency operators with AND, included more keyword terms	412	53	53.5%	12.9%	13812	61	61.6%	0.4%
Reproductive coercion [28]	Scoping Review	28	Ovid Medline	Replaced adjacency operators with AND	904	4	14.3%	0.4%	2448	6	21.4%	0.2%
ED flow training [29]	Integrative Review	46	Ovid Medline	Removed concept group relating to interventions that improve flow	2565	38	82.6%	1.5%	4753	40	87.0%	0.8%
AI ethics [30]	Scoping Review	43	Scopus	Focused education concept groups to include postsecondary settings only	18663	36	83.7%	0.2%	9561	28	65.1%	0.3%

Since the reviews evaluated in the present study used a multi-pronged search strategy and included multiple databases, it was possible for the expanded database search strategy to retrieve more included articles than the original search. For the quantitative and qualitative systematic reviews (n = 6), all of the included articles retrieved by the revised search were also captured in the original search. However, this was not the case for the scoping and integrative reviews, where the broadened search retrieved more articles than were included in the original search. Similarly, the narrowed AI ethics search retrieved fewer articles than were included in the original search (see Table 1).

RIS files for each search were downloaded, and each citation was labelled as included ("relevant") or excluded ("irrelevant") based on the list of studies included in the completed review. The labelled RIS file for each search was imported into the ASReview web app in simulation mode. Before a simulation is initiated, ASReview also requires that one relevant and one irrelevant article be identified to prime the model. The first randomly generated relevant and irrelevant article were selected for each simulation. The recommended default automated screening settings were used (Feature extraction technique: Term Frequency-Inverse Document Frequency; Classifier: Naive Bayes; Query strategy: Maximum; Balance strategy: Dynamic resampling, double). Once the simulation is started, articles are re-prioritized automatically and continuously based on what the software learns from each “relevant” or “irrelevant” label that it encounters. Each search simulation was run ten times in order to account for variance introduced by the articles used to prime the model.

Recall data from the completed simulation runs were downloaded and analyzed. Sample graphical and numerical data output from the ASReview LAB simulation module are available in Appendix B.

The number of results that needed to be screened to achieve recall of 95% or above for all included articles retrieved by the database search was determined for each simulation, with the results from each search averaged together. A 95% recall level is often used in machine-learning screening tool simulation studies, as it approximates rates of human error during screening [31,32]. In addition, the total screening time for each search was estimated using a rate of 1 record screened per minute [33]. This estimate is commonly cited in studies evaluating the performance of semi-automated screening tools.

In real-world review scenarios, the true number of included articles in any given result set is unknown prior to full-text screening. Accordingly, I also evaluated the number of results screened, included articles retrieved, and recall level when three different stopping rules were applied. Recall levels are difficult to compare for those projects where broader searches retrieved more included articles. In order to manage this issue, I also calculated an adjusted recall value that reflected all potentially relevant articles that could have been retrieved in the database, not just those that were retrieved by the narrower search.

Callaghan and Müller-Hansen identify a number of different types of stopping rules, including statistical, heuristic, automatic and pragmatic [34]. For the purposes of this study, I selected three stopping rules that were straightforward to perform and explain:

1. A data-based heuristic (Ros et al. 2017): Screening stops after *n* consecutive irrelevant records [35]. König et al. report that the value selected for *n* in published studies varied from 20-500 [36]. For the purposes of this study, I chose 50 irrelevant records.
2. A time-based heuristic (Wallace et al. 2010): Screening stops after 50% of the total number of records are screened [8].
3. A mixed heuristic called the SAFE procedure (Boetje and van de Schoot 2024): Screening stops when four independent conditions are satisfied, including one where a crude estimate of relevant records in the total dataset is calculated (the RR_T) [37].

For this study, I modified the SAFE procedure to stop screening when the following conditions were met: 1) twice the estimated number of relevant records were screened (2 x RR_T); to calculate this number, I followed the outlined estimation procedures which included random sampling of the citation dataset 2) a minimum of 10% of the records were screened; and 3) 50 consecutive irrelevant records were identified. The original SAFE procedure also requires that all key papers be identified; however, this criterion was excluded as selecting key papers in a retrospective study is challenging and likely to introduce bias.

## RESULTS

For systematic review projects, the absolute increase in number of records required to be screened to reach 95% recall between narrower searches and broader searches was minimal in 4 out of 6 cases (see Table 2). Here, “absolute increase” refers to the raw difference in record counts, whereas “relative increase” expresses that difference as a percentage. Assuming a rate of 1 record screened per minute by a single reviewer, the time difference between broader and narrower searches was ≤35 minutes for the four quantitative reviews. In contrast, the impact was more pronounced in the qualitative reviews: the broader search for the autism diagnosis review required an additional 1 hour and 45 minutes of screening time to reach 95% recall, while the chronic illness and workplace policy review required 8 hours and 46 minutes more. If dual, independent screening is conducted, the broader search for the chronic illness & workplace policy review would result in an additional 17.5 hours of screening time.

**Table 2:** Average number of records screened to reach recall of at least 95%.

Project name	Review Type	Narrower search	Broader Search	Absolute increase (# of articles)	Relative Increase (%)
SGLT2 safety	Quant SR	225	247	22	9.6
Chronic pain peer support	Quant SR	159	185	26	16.3
Cannabis purchase choice	Quant SR	268	303	35	13.1
Production effect	Quant SR	125	149	24	19.5
Autism diagnosis experiences	Qual SR	342	447	105	30.7
Chronic illness & workplace policy	Qual SR	356	883	527	147.7

It is more difficult to compare the differences in workload between narrower and broader searches for the scoping reviews and integrative review, as the broader searches also retrieved more included articles. For the scoping reviews, the absolute increase in screening time for a single reviewer ranged from 2 hours and 20 minutes to almost 30 hours. The relative increase in screening burden for the scoping reviews ranged from 327-875% (see Table 3). The difference in workload between the narrower and broader searches for the integrative review was less pronounced.

**Table 3:** Average number of records screened to reach recall of at least 95%.

Project name	Review Type	Narrower search	Broader search	Absolute increase (# of articles)	Relative increase (%)	Additional articles retrieved
ICU sustainability	Scoping	120	557	437	364.2	8
Reproductive coercion	Scoping	16	156	140	875.0	2
ED flow training	Integrative	997	1107	110	11.0	2
AI ethics	Scoping	549	2346	1797	327.3	8

When examining the effectiveness and efficiency of broader and narrower searches when stopping rules were used, several trends emerge. First, many of the narrower and broader searches failed to reach 95% recall when the “50 consecutive irrelevant records” stopping rule was applied, although the absolute difference in the number of records screened between narrow and broad searches was minimal (Table 4). Second, recall was higher when employing the “screening 50% of records” stopping rule; all searches attained 95% recall or above when unadjusted (Table 5). As expected, though, this stopping rule substantially increases the time to screening completion for broader searches. In the case of the ICU sustainability project, for example, time to completion of screening by a single reviewer would increase by over 110 hours. Finally, under the SAFE stopping procedure, broader searches achieved higher recall with smaller differences in absolute number of articles screened (Table 6). Nevertheless, 3 out of 10 broad searches still failed to meet the 95% recall threshold.

**Table 4:** Average number of records screened, included articles retrieved, and recall when applying “50 consecutive irrelevant records” stopping rule.

Project name	Records screened		Included articles retrieved		Recall (adjusted)	
	Narrow database search	Broad database search	Narrow database search	Broad database search	Narrow database search	Broad database search
SGLT2 safety	320	365	104	104	99.0%	99.0%
Chronic pain peer support	144	126	11	11	68.8%	68.8%
Cannabis purchase choice	118	116	16	15	71.7%	67.0%
Production effect	121	127	15	15	95.0%	92.5%
Autism diagnosis experiences	146	193	12	13	75.0%	79.4%
Chronic illness & workplace policy	253	237	19	16	78.3%	67.1%
ICU sustainability	177	285	52	54	97.7% (85.0%)	87.9%
Reproductive coercion	65	90	4	5	100% (66.7%)	85.0%
ED flow training	198	188	18	18	46.3% (44.0%)	44.8%
AI ethics	213	309	17	21	59.3% (46.1%)	59.2%

**Table 5:** Average number of records screened, included articles retrieved, and recall when applying “50% of total number of records” stopping rule.

Project name	Records screened		Included articles retrieved		Recall (adjusted)	
	Narrow database search	Broad database search	Narrow database search	Broad database search	Narrow database search	Broad database search
SGLT2 safety	386	855	105	105	100.0%	100.0%
Chronic pain peer support	1337	1656	16	16	100.0%	100.0%
Cannabis purchase choice	711	1955	23	23	100.0%	100.0%
Production effect	235	779	16	16	100.0%	100.0%
Autism diagnosis experiences	624	1865	16	16	100.0%	100.0%
Chronic illness & workplace policy	368	1939	23	24	95.4%	100.0%
ICU sustainability	206	6906	53	61	99.1% (86.1%)	100.0%
Reproductive coercion	452	1224	4	6	100% (66.7%)	100.0%
ED flow training	1283	2377	38	39	100% (95.0%)	97.5%
AI ethics	4797	9332	28	35	100% (77.8%)	97.2%

**Table 6:** Average number of records screened, included articles retrieved, and recall when using modified SAFE procedure.

Project name	Records screened		Included articles retrieved		Recall (adjusted)	
	Narrow database search	Broad database search	Narrow database search	Broad database search	Narrow database search	Broad database search
SGLT2 safety	320	365	104	104	99.0%	99.0%
Chronic pain peer support	267	331	16	16	100.0%	100.0%
Cannabis purchase choice	247	391	21	22	90.9%	95.7%
Production effect	121	187	15	16	95.0%	98.8%
Autism diagnosis experiences	186	373	14	15	88.1%	93.8%
Chronic illness & workplace policy	253	394	19	20	78.3%	84.2%
ICU sustainability	177	1381	52	61	97.7% (85.9%)	100.0%
Reproductive coercion	200	245	4	6	100% (66.7%)	100.0%
ED flow training	340	507	27	31	72.1% (68.5%)	77.3%
AI ethics	959	1866	28	34	100% (77.7%)	95.3%

## DISCUSSION

A range of complex factors impact screening workload and recall when active learning screening tools are used in evidence synthesis projects. The most widely studied factor is the screening tool itself [17,31,38–50]. The impact of different algorithmic models has received some attention [51–53], and two studies have evaluated the performance of various stopping rules [36,54]. However, the influence of dataset-related characteristics remains relatively underexplored. While some non-comparative studies have presented screening outcomes relating to specific topics [55–58] or types of evidence synthesis projects [59,60], features of the dataset associated with search sensitivity and precision have not been empirically studied. Moreover, few studies have examined the relationship among these factors, although König et al. [36] have noted interactions between “the performance of the stopping rules and the number of relevant studies” in the dataset.

Given that our understanding of how various factors interact to affect screening workload and recall is still evolving, recommendations to increase search sensitivity when using active learning screening tools may be premature. In this study, broader searches for quantitative systematic reviews tended to achieve high recall with minimal differences in absolute number of records screened. However, differences in workload and recall between narrower and broader searches were more pronounced for qualitative systematic reviews. Semi-automated screening of the broader chronic illness and workplace policy search would require more time than a full manual screening of the narrower result set. In other review types such as scoping and integrative reviews, broader searches retrieved more articles but also imposed substantial screening burdens.

These findings suggest that librarians and information specialists should consider multiple factors when determining the appropriate balance between search sensitivity and specificity in the context of semi-automated screening tools. For example, selecting a sensitive methods filter may not increase workload for quantitative systematic reviews of interventions. In contrast, a narrower search strategy supplemented by snowball searching [61] may prove more time-efficient for “fuzzy” topics or other types of reviews. In addition, it is important to consider the impact of stopping rules. Although screening 50% of the retrieved records yielded the highest recall among the stopping rules tested, applying this threshold in conjunction with broader searches led to considerable increases in screening workload.

## LIMITATIONS

Although this study offers preliminary insights into the impact of search sensitivity and specificity in the context of semi-automated screening, its generalizability is limited by the small number of reviews analyzed. Consequently, definitive conclusions cannot be drawn from the findings.

It should also be noted that a single database was selected for each review, a choice that may bias screening workload results downwards. Furthermore, “included articles” were classified as those that were retained at the final stage of the review project. An alternative approach would have been to label articles retained after the title and abstract screening stage as “included”. The implications of this decision are mixed. On one hand, restricting inclusion to final-stage articles reduces noise in the dataset and may result in relevant articles being identified more quickly. On the other hand, the resulting reduction in data points may have meant that it was more difficult for the software to learn what was relevant or irrelevant, particularly for reviews with few included articles.

Finally, the stopping rules used in this study were relatively simple to perform. The use of more sophisticated statistical stopping rules may have enhanced both recall and precision.

## Data Availability

Data associated with this article are available via the Memorial University Dataverse: https://doi.org/10.5683/SP3/TOLDSZ.

## SUPPLEMENTAL FILES

**Appendix A:** Original and Revised Search Strategies

**Appendix B:** Figures, Etc.
