# Supplementary material for: Comparing the performance of narrow vs. broad search strategies when using machine learning-based software for title/abstract screening
Source: J Med Libr Assoc. 2026 Apr 13;114(2):105–15. doi: 10.5195/jmla.2026.2286 (PMC13075572; doi:10.5195/jmla.2026.2286)
Supplement: Supplementary file 1 — Appendix A: Original and Revised Search Strategies [file jmla-114-2-105-s01.docx]

**Appendix I – Original and revised search strategies**

**SGLT2 safety - original PubMed search strategy**

|  | **Search String** |
| --- | --- |
| #1 | "Diabetes Mellitus, Type 2"[Mesh] OR NIDDM[tw] OR t2dm[tw] OR ((“type 2"[tw] OR "type ii"[tw] OR "adult onset"[tw] OR “mature onset”[tw] OR “late onset”[tw] OR "noninsulin-dependent”[tw] OR “non insulin dependent”[tw]) AND diabetes[tw]) |
| #2 | "Sodium-Glucose Transport Proteins/antagonists and inhibitors"[Mesh] OR "Sodium-Glucose Transporter 2"[Mesh] OR "sodium-glucose co-transporter 2"[tw] OR SGL2[tw] OR SGLT2[tw] OR gliflozin*[tw] OR "Canagliflozin"[Mesh] OR canagliflozin*[tw] OR invokana[tw] OR sulisent[tw] OR "TA 7284"[tw] OR TA7284[tw] OR "JNJ 28431754"[tw] OR JNJ28431754[tw] OR "2-(3-(4-ethoxybenzyl)-4-chlorophenyl)-6-hydroxymethyltetrahydro-2H-pyran-3,4,5-triol"[Supplementary Concept] OR dapagliflozin*[tw] OR farxiga[tw] OR forxiga[tw] OR “BMS 512148”[tw] OR BMS512148[tw] OR "empagliflozin"[Supplementary Concept] OR empagliflozin*[tw] OR jardiance[tw] OR "BI 10773"[tw] OR BI10773[tw] OR ipragliflozin[Supplementary Concept] OR ipragliflozin*[tw] OR suglat[tw] OR "ASP 1941"[tw] OR ASP1941[tw] OR "1,5-anhydro-1-(5-(4-ethoxybenzyl)-2-methoxy-4-methylphenyl)-1-thioglucitol"[Supplementary Concept] OR luseogliflozin*[tw] OR lusefi[tw] OR “TS 071”[tw] OR TS071[tw] OR "remogliflozin etabonate"[Supplementary Concept] OR remogliflozin*[tw] OR "KGT 1681"[tw] OR KGT1681[tw] OR "(2S,3R,4R,5S,6R)-2-(4-chloro-3-(4-ethoxybenzyl)phenyl)-6-(methylthio)tetrahydro-2H-pyran-3,4,5-triol" [Supplementary Concept] OR sotagliflozin*[tw] OR "LX 4221"[tw] OR LX4221[tw] OR "6-((4-ethylphenyl)methyl)-3',4',5',6'-tetrahydro-6'-(hydroxymethyl)spiro(isobenzofuran-1(3H),2'-(2H)pyran)-3',4',5'-triol" [Supplementary Concept] OR tofogliflozin*[tw] OR apleway[tw] OR deberza[tw] OR "CSG 452"[tw] OR CSG452[tw] OR "5-(4-chloro-3-(4-ethoxybenzyl)phenyl)-1-hydroxymethyl-6,8-dioxabicyclo(3.2.1)octane-2,3,4-triol" [Supplementary Concept] OR ertugliflozin*[tw] OR "PF 04971729"[tw] OR PF04971729[tw] |
| #3 | ("randomized controlled trial"[pt] OR "controlled clinical trial"[pt] OR randomized[tiab] OR placebo[tiab] OR "clinical trials as topic"[Mesh:NoExp] OR randomly[tiab] OR trial[ti]) NOT (animals[mh] NOT humans[mh]) |
| #4 | #1 AND #2 AND #3 |

**SGLT2 safety - revised PubMed search strategy**

|  | **Search String** |
| --- | --- |
| #1 | "Diabetes Mellitus, Type 2"[Mesh] OR NIDDM[tw] OR t2dm[tw] OR ((“type 2"[tw] OR "type ii"[tw] OR "adult onset"[tw] OR “mature onset”[tw] OR “late onset”[tw] OR "noninsulin-dependent”[tw] OR “non insulin dependent”[tw]) AND diabetes[tw]) |
| #2 | "Sodium-Glucose Transport Proteins/antagonists and inhibitors"[Mesh] OR "Sodium-Glucose Transporter 2"[Mesh] OR "sodium-glucose co-transporter 2"[tw] OR SGL2[tw] OR SGLT2[tw] OR gliflozin*[tw] OR "Canagliflozin"[Mesh] OR canagliflozin*[tw] OR invokana[tw] OR sulisent[tw] OR "TA 7284"[tw] OR TA7284[tw] OR "JNJ 28431754"[tw] OR JNJ28431754[tw] OR "2-(3-(4-ethoxybenzyl)-4-chlorophenyl)-6-hydroxymethyltetrahydro-2H-pyran-3,4,5-triol"[Supplementary Concept] OR dapagliflozin*[tw] OR farxiga[tw] OR forxiga[tw] OR “BMS 512148”[tw] OR BMS512148[tw] OR "empagliflozin"[Supplementary Concept] OR empagliflozin*[tw] OR jardiance[tw] OR "BI 10773"[tw] OR BI10773[tw] OR ipragliflozin[Supplementary Concept] OR ipragliflozin*[tw] OR suglat[tw] OR "ASP 1941"[tw] OR ASP1941[tw] OR "1,5-anhydro-1-(5-(4-ethoxybenzyl)-2-methoxy-4-methylphenyl)-1-thioglucitol"[Supplementary Concept] OR luseogliflozin*[tw] OR lusefi[tw] OR “TS 071”[tw] OR TS071[tw] OR "remogliflozin etabonate"[Supplementary Concept] OR remogliflozin*[tw] OR "KGT 1681"[tw] OR KGT1681[tw] OR "(2S,3R,4R,5S,6R)-2-(4-chloro-3-(4-ethoxybenzyl)phenyl)-6-(methylthio)tetrahydro-2H-pyran-3,4,5-triol" [Supplementary Concept] OR sotagliflozin*[tw] OR "LX 4221"[tw] OR LX4221[tw] OR "6-((4-ethylphenyl)methyl)-3',4',5',6'-tetrahydro-6'-(hydroxymethyl)spiro(isobenzofuran-1(3H),2'-(2H)pyran)-3',4',5'-triol" [Supplementary Concept] OR tofogliflozin*[tw] OR apleway[tw] OR deberza[tw] OR "CSG 452"[tw] OR CSG452[tw] OR "5-(4-chloro-3-(4-ethoxybenzyl)phenyl)-1-hydroxymethyl-6,8-dioxabicyclo(3.2.1)octane-2,3,4-triol" [Supplementary Concept] OR ertugliflozin*[tw] OR "PF 04971729"[tw] OR PF04971729[tw] |
| #3 | ("randomized controlled trial"[pt] OR "controlled clinical trial"[pt] OR randomized[tiab] OR placebo[tiab] OR "drug therapy"[sh] OR randomly[tiab] OR trial[tiab] OR groups[tiab]) NOT (animals[mh] NOT humans[mh]) |
| #4 | #1 AND #2 AND #3 |

**Chronic pain peer support – original embase.com search strategy**

| No. | Query |
| --- | --- |
| #36 | #35 AND [01-01-1900]/sd NOT [29-05-2024]/sd |
| #35 | #34 AND ([english]/lim OR [french]/lim) |
| #34 | #32 NOT #33 |
| #33 | cancer:ti,ab NOT ('non cancer':ti,ab OR noncancer:ti,ab) |
| #32 | #19 AND #31 |
| #31 | #23 OR #24 OR #25 OR #26 OR #27 OR #28 OR #29 OR #30 |
| #30 | ((online OR internet OR virtual OR 'social media' OR 'message board*' OR facebook OR twitter OR instagram OR reddit OR tiktok OR snapchat OR myspace OR douyin OR kuaishou OR weibo) NEAR/3 support*):ti,ab |
| #29 | 'expert patient program*':ti,ab |
| #28 | 'support group'/exp OR 'support group$':ti,ab |
| #27 | peer$:ti,ab AND 'self management':ti,ab |
| #26 | (lay NEAR/2 (mentor* OR coach* OR volunteer$ OR led OR delivered OR run OR directed)):ti,ab |
| #25 | (peer$ NEAR/2 (mentor* OR coach* OR volunteer$ OR led OR delivered OR run OR directed)):ti,ab |
| #24 | (peer$ NEAR/3 support$):ti,ab |
| #23 | #20 AND (#21 OR #22) |
| #22 | 'self management':ti,ab |
| #21 | 'peer group'/de OR peer$:ti,ab |
| #20 | 'social support'/mj |
| #19 | #1 OR #2 OR #3 OR #4 OR #5 OR #6 OR #7 OR #8 OR #9 OR #10 OR #11 OR #12 OR #13 OR #14 OR #15 OR #16 OR #17 OR #18 |
| #18 | 'trigeminus neuralgia'/de OR 'trigeminal neuralgia':ti,ab OR 'tic douloureux':ti,ab |
| #17 | 'somatoform disorder'/de OR 'somatic symptom disorder':ti,ab |
| #16 | 'sciatica'/exp OR sciatica:ti,ab |
| #15 | 'multiple sclerosis'/exp OR 'multiple sclerosis':ti,ab |
| #14 | 'myofascial pain'/de |
| #13 | headache*:ti,ab OR 'head ache*':ti,ab |
| #12 | 'migraine'/de OR migraine:ti,ab |
| #11 | 'systemic lupus erythematosus'/exp OR lupus:ti,ab |
| #10 | 'fibromyalgia'/de OR fibromyalgia:ti,ab |
| #9 | 'endometriosis'/exp OR endometriosis:ti,ab |
| #8 | 'ehlers danlos syndrome'/exp OR 'ehlers danlos':ti,ab |
| #7 | 'complex regional pain syndrome'/de |
| #6 | 'interstitial cystitis'/exp OR 'interstitial cystitis':ti,ab |
| #5 | 'central nervous system sensitization'/exp OR 'central nervous system sensitization' |
| #4 | 'rheumatoid arthritis'/exp OR 'psoriatic arthritis'/exp OR 'rheumatic polymyalgia'/de OR arthritis:ti,ab OR rheumati*:ti,ab OR spondyloarthritis:ti,ab OR osteoarthritis:ti,ab OR 'sjogren* syndrome':ti,ab OR 'systemic sclerosis':ti,ab |
| #3 | 'sickle cell anemia'/exp OR 'sickle cell an$emia':ti,ab |
| #2 | 'amputee'/de OR amputee$:ti,ab OR 'limb loss':ti,ab |
| #1 | 'pain'/mj OR 'chronic pain'/exp OR pain:ti,ab |

**Chronic pain peer support – revised embase.com search strategy**

| No. | Query |
| --- | --- |
| #36 | #35 AND [01-01-1900]/sd NOT [29-05-2024]/sd |
| #35 | #34 AND ([english]/lim OR [french]/lim) |
| #34 | #32 NOT #33 |
| #33 | cancer:ti,ab NOT ('non cancer':ti,ab OR noncancer:ti,ab) |
| #32 | #19 AND #31 |
| #31 | #23 OR #24 OR #25 OR #26 OR #27 OR #28 OR #29 OR #30 |
| #30 | ((online OR internet OR virtual OR 'social media' OR 'message board*' OR facebook OR twitter OR instagram OR reddit OR tiktok OR snapchat OR myspace OR douyin OR kuaishou OR weibo) NEAR/3 support*):ti,ab |
| #29 | 'expert patient program*':ti,ab |
| #28 | 'support group'/exp OR 'support group$':ti,ab |
| #27 | peer$:ti,ab AND 'self management':ti,ab |
| #26 | (lay NEAR/2 (mentor* OR coach* OR volunteer$ OR led OR delivered OR run OR directed)):ti,ab |
| #25 | (peer$ NEAR/2 (mentor* OR coach* OR volunteer$ OR led OR delivered OR run OR directed)):ti,ab |
| #24 | (peer$ NEAR/3 support$):ti,ab |
| #23 | #20 AND (#21 OR #22) |
| #22 | 'self management':ti,ab |
| #21 | 'peer group'/de OR peer$:ti,ab |
| #20 | 'social support'/de |
| #19 | #1 OR #2 OR #3 OR #4 OR #5 OR #6 OR #7 OR #8 OR #9 OR #10 OR #11 OR #12 OR #13 OR #14 OR #15 OR #16 OR #17 OR #18 |
| #18 | 'trigeminus neuralgia'/de OR 'trigeminal neuralgia':ti,ab OR 'tic douloureux':ti,ab |
| #17 | 'somatoform disorder'/de OR 'somatic symptom disorder':ti,ab |
| #16 | 'sciatica'/exp OR sciatica:ti,ab |
| #15 | 'multiple sclerosis'/exp OR 'multiple sclerosis':ti,ab |
| #14 | 'myofascial pain'/de |
| #13 | headache*:ti,ab OR 'head ache*':ti,ab |
| #12 | 'migraine'/de OR migraine:ti,ab |
| #11 | 'systemic lupus erythematosus'/exp OR lupus:ti,ab |
| #10 | 'fibromyalgia'/de OR fibromyalgia:ti,ab |
| #9 | 'endometriosis'/exp OR endometriosis:ti,ab |
| #8 | 'ehlers danlos syndrome'/exp OR 'ehlers danlos':ti,ab |
| #7 | 'complex regional pain syndrome'/de |
| #6 | 'interstitial cystitis'/exp OR 'interstitial cystitis':ti,ab |
| #5 | 'central nervous system sensitization'/exp OR 'central nervous system sensitization' |
| #4 | 'rheumatoid arthritis'/exp OR 'psoriatic arthritis'/exp OR 'rheumatic polymyalgia'/de OR arthritis:ti,ab OR rheumati*:ti,ab OR spondyloarthritis:ti,ab OR osteoarthritis:ti,ab OR 'sjogren* syndrome':ti,ab OR 'systemic sclerosis':ti,ab |
| #3 | 'sickle cell anemia'/exp OR 'sickle cell an$emia':ti,ab |
| #2 | 'amputee'/de OR amputee$:ti,ab OR 'limb loss':ti,ab |
| #1 | 'pain'/exp OR pain:ti,ab |

**Cannabis purchase choice – original & revised Ovid Medline search strategy**

1 marijuana smoking/ 5784

2 "marijuana use"/ 2169

3 ((marijuana or marihuana or cannabis or bhang or charas or ganja or hash or hashish or pot or dope or weed or dab or dabs or kief or shatter or budder or CBD or THC or cannabidiol) adj3 use*).ti,ab. 25352

4 ((marijuana or marihuana or cannabis or bhang or charas or ganja or hash or hashish or pot or dope or weed or dab or dabs or kief or shatter or budder) adj2 smok*).ti,ab. 2928

5 ((marijuana or marihuana or cannabis or bhang or charas or ganja or hash or hashish or pot or dope or weed or dab or dabs or kief or shatter or budder or CBD or THC or cannabidiol) adj oil*).ti,ab. 434

6 ((marijuana or marihuana or cannabis or bhang or charas or ganja or hash or hashish or pot or dope or weed or dab or dabs or kief or shatter or budder or CBD or THC or cannabidiol) adj edible*).ti,ab. 106

7 ((marijuana or marihuana or cannabis or bhang or charas or ganja or hash or hashish or pot or dope or weed or dab or dabs or kief or shatter or budder or CBD or THC or cannabidiol) and consumer*).ti,ab. 1047

8 ((marijuana or marihuana or cannabis or bhang or charas or ganja or hash or hashish or pot or dope or weed or dab or dabs or kief or shatter or budder or CBD or THC or cannabidiol) and customer*).ti,ab. 56

9 (recreational adj (cannabis or marijuana or marihuana)).ti,ab. 1231

10 or/1-9 29372

11 commerce/ 30862

12 ec.fs. 451178

13 economic*.mp. 853637

14 exp "Costs and Cost Analysis"/ 275908

15 cost*.mp. 981359

16 afford*.mp. 148510

17 (price or prices or pricing).mp. 57578

18 quality.mp. 1768486

19 aroma*.mp. 181777

20 scent*.mp. 5417

21 taste*.mp. 58120

22 (flavor* or flavour*).mp. 38154

23 wet*.mp. 140924

24 dry*.mp. 261544

25 humidity.mp. 57078

26 potency.mp. 129758

27 (thc adj2 level*).mp. 383

28 (thc adj2 percent*).mp. 31

29 (thc adj2 ratio*).mp. 134

30 (cbd adj2 level*).mp. 172

31 (cbd adj2 percent*).mp. 12

32 (cbd adj2 ratio*).mp. 142

33 (cannabidiol adj2 level*).mp. 48

34 (cannabidiol adj2 percent*).mp. 3

35 (cannabidiol adj2 ratio*).mp. 35

36 cannabinoid profile*.mp. 87

37 terpene profile*.mp. 107

38 strain*.mp. 1192436

39 sativa.mp. 35969

40 indica.mp. 12261

41 hybrid.mp. 252899

42 (product adj selection).mp. 526

43 warning label*.mp. 1443

44 warning lable*.mp. 1

45 warning message*.mp. 345

46 packaging.mp. 44774

47 legal.mp. 139185

48 illegal.mp. 14887

49 dispensar*.mp. 5921

50 illicit.mp. 30616

51 (black adj market).mp. 428

52 dealer*.mp. 1098

53 (delivery or deliveries).mp. 821949

54 (local adj (shop or dispensar* or business* or producer*)).mp. 404

55 (independent adj (shop or dispensar* or business* or producer*)).mp. 54

56 (small adj (shop or dispensar* or business* or producer*)).mp. 1525

57 or/11-56 6056445

58 Patient Preference/ 11668

59 Economics, Behavioral/ 874

60 Choice Behavior/ 36114

61 exp attitude/ 664530

62 Decision Making/ 108651

63 preference*.mp. 231951

64 choice*.mp. 471457

65 attitude*.mp. 512019

66 decision*.mp. 663509

67 perception*.mp. 572665

68 ((economic or consumer or customer or user) and (behavior* or behaviour*)).mp. 93902

69 practice*.mp. 1578846

70 substitut*.mp. 453596

71 purchase task*.mp. 294

72 demand.mp. 233146

73 incentiv*.mp. 49893

74 elasticity.mp. 74003

75 or/58-74 4233428

76 10 and 57 and 75 2628

77 Animal/ not Human/ 5265353

78 76 not 77 2591

79 limit 78 to english 2492

80 limit 79 to dt=19600101-20200411 1421 (original)

81 10 and 75 6348

82 81 not 77 6247

83 limit 82 to dt=19600101-20200411 3909 (revised)

**Production effect – original & revised APA PsycInfo search strategy**

| **#** | **Query** | **Results** |
| --- | --- | --- |
| S1 | TI "production effect#" OR AB "production effect#" OR KW "production effect#" OR TI "pronunciation effect#" OR AB "pronunciation effect#" OR KW "pronunciation effect#" | 189 |
| S2 | TI ( (word or list) N2 (retention OR retain* OR memor*) ) OR AB ( (word or list) N2 (retention OR retain* OR memor*) ) | 5,489 |
| S3 | TI ( pronounc* OR vocali* OR voice* OR verbal* OR aloud OR speak* OR spoken OR speech OR whisper* ) OR AB ( pronounc* OR vocali* OR voice* OR verbal* OR aloud OR speak* OR spoken OR speech OR whisper* ) | 369,035 |
| S4 | DE "Between Groups Design" OR DE "Experimental Subjects" | 5,834 |
| S5 | TI ( ( between OR independent ) N2 (group# OR subject# OR design#) ) OR AB ( between OR independent ) N2 (group# OR subject# OR design#) ) | 126,223 |
| S6 | TI (pure N2 list#) OR AB (pure N2 list#) | 172 |
| S7 | TI experiment# OR AB experiment# | 284,463 |
| S8 | S4 OR S5 OR S6 OR S7 | 407,345 |
| S9 | S2 AND S3 AND S8 | 387 |
| S10 | S1 OR S9 | 558 |
| S11 | RD 19000101-20210915 | 5,058,440 |
| S12 | S10 AND S11 (original) | 469 |
| S13 | S1 OR (S2 AND S3) | 1,772 |
| S14 | S11 AND S13 (revised) | 1,557 |

**Autism diagnosis experiences – original & revised CINAHL Plus search strategy**

| **#** | **Query** | **Results** |
| --- | --- | --- |
| S1 | MH "Autistic Disorder" OR MH "Asperger Syndrome" OR TI autism OR TI autistic OR TI asperger* OR AB autism OR AB autistic OR AB asperger* | 35,062 |
| S2 | MH "Parents" OR MH "Adoptive Parents" OR MH "Foster Parents" OR MH "Fathers" OR MH "Mothers" OR TI parent* OR TI father* OR TI mother* OR TI guardian* OR AB parent* OR AB father* OR AB mother* OR AB guardian* | 294,552 |
| S3 | MH "Diagnosis+" OR MW "di" OR TI diagnos* OR AB diagnos* | 2,757,808 |
| S4 | MH "Qualitative Studies+" OR MH "Phenomenology" OR MH "Audiorecording" OR MH "Focus Groups" OR MH "Interviews+" OR MH "Narratives" OR MH "Observational Methods+" OR MH "Life Experiences" OR MH "Thematic Analysis" OR MH "Parental Attitudes+" OR MH "Health Beliefs" OR TI qualitative OR AB qualitative OR TI interview* OR AB interview* OR TI "mixed method" OR AB "mixed method" OR TI "mixed methods" OR AB "mixed methods" | 614,760 |
| S5 | S1 AND S2 AND S3 AND S4 | 1,476 |
| S6 | EM 19000101-20230606 | 7,130,365 |
| S7 | S5 AND S6 (original) | 1,252 |
| S8 | S1 AND S2 AND S3 | 4,346 |
| S9 | S6 AND S8 (revised) | 3,734 |

**Chronic illness & workplace policy – original & revised CINAHL Plus search strategy**

| **#** | **Query** | **Results** |
| --- | --- | --- |
| S1 | MH "Chronic Disease" OR TI "chronic disease*" OR AB "chronic disease*" OR TI "chronic* ill*" OR AB "chronic* ill*" OR TI "chronic condition*" OR AB "chronic condition*" | 114,386 |
| S2 | MH "Arthritis+" OR TI arthritis OR AB arthritis OR MH "Asthma+" OR TI asthma OR AB asthma OR MH "Cancer Patients" OR TI cancer OR AB cancer OR MH "Neoplasms+" OR MH "Cardiovascular Diseases+" OR MH "Cerebrovascular Disorders+" OR TI "coronary artery disease" OR AB "coronary artery disease" OR MH "Stroke Patients" OR TI stroke OR AB stroke OR MH "Renal Insufficiency, Chronic+" OR TI "chronic kidney" OR AB "chronic kidney" OR MH "Dialysis Patients" OR MH "Fatigue Syndrome, Chronic" OR TI "chronic fatigue syndrome" OR AB "chronic fatigue syndrome" OR MH "Pulmonary Disease, Chronic Obstructive+" OR TI COPD OR AB COPD OR TI "chronic obstructive pulmonary disease" OR AB "chronic obstructive pulmonary disease" OR MH "Crohn Disease" OR TI crohn* OR AB crohn* OR MH "Diabetes Mellitus+" OR MH "Diabetic Patients" OR TI diabet* OR AB diabet* OR MH "Epilepsy+" OR TI epilepsy OR AB epilepsy OR MH "Fibromyalgia" OR TI fibromyalgia OR AB fibromyalgia OR MH "Hepatitis+" OR TI hepatitis OR AB hepatitis OR MH "Human Immunodeficiency Virus+" OR MH "HIV Infections+" OR MH "HIV-Infected Patients+" OR TI HIV OR AB HIV OR TI AIDS OR AB AIDS OR MH "Lupus Erythematosus, Systemic+" OR TI "systemic lupus" OR AB "systemic lupus" OR MH "Amyotrophic Lateral Sclerosis" OR TI ALS OR AB ALS OR TI "amyotrophic lateral sclerosis" OR AB "amyotrophic lateral sclerosis" OR TI "lou gehrig*" OR AB "lou gehrig*" OR MH "Multiple Sclerosis+" OR TI "multiple sclerosis" OR AB "multiple sclerosis" OR MH "Parkinson Disease" OR TI parkinson* OR AB parkinson* OR MH "Colitis, Ulcerative" OR TI "ulcerative colitis" OR AB "ulcerative colitis" | 2,223,515 |
| S3 | S1 OR S2 | 2,292,416 |
| S4 | (TI employment OR TI labor OR TI labour OR TI work OR TI workplace) N2 (TI policy OR TI policies OR TI legislat* OR TI law OR TI laws OR TI regulation*) | 1,207 |
| S5 | (AB employment OR AB labor OR AB labour OR AB work OR AB workplace) N1 (AB policy OR AB policies OR AB legislat* OR AB law OR AB laws OR AB regulation*) | 2,458 |
| S6 | (MH "Employment" OR MH "Job Market" OR MH "Work") AND (MH "Organizational Policies+" OR MH "Government Regulations" OR MH "Public Policy" OR MH "Legislation, Labor" OR MW "lj" OR MW "st") | 4,401 |
| S7 | MH "Employment of Disabled" OR MH "Employment, Supported" OR MH "Employee Rights" OR MH "Job Accommodation" OR TI "job accommodation*" OR AB "job accommodation*" OR TI "work* accommodation*" OR AB "work* accommodation*" OR MH "Rehabilitation, Vocational" OR MH "Insurance, Disability+" OR TI "disability insurance" OR AB "disability insurance" OR TI "incapacity benefit*" OR AB "incapacity benefit*" OR TI "disability benefit*" OR AB "disability benefit*" OR MH "Job Re-Entry" OR TI "job re-entry" OR AB "job re-entry" OR TI "job reentry" OR AB "job reentry" OR TI "return to work" OR AB "return to work" OR MH "Family and Medical Leave" OR MH "Sick Leave" OR TI "sick leave" OR AB "sick leave" OR TI "medical leave" OR AB "medical leave" OR TI "absence polic*" OR AB "absence polic*" OR MH "Health Benefit Plans, Employee" OR TI "benefit plan*" OR AB "benefit plan*" OR MM "Public Assistance" OR TI "public assistance" OR AB "public assistance" OR TI "social assistance" OR AB "social assistance" OR MM "Social Welfare" OR MM "Economic and Social Support" OR MM "Unemployment" | 41,578 |
| S8 | S4 OR S5 OR S6 OR S7 | 47,933 |
| S9 | MH "Qualitative Studies+" OR MH "Phenomenology" OR MH "Audiorecording" OR MH "Focus Groups" OR MH "Interviews+" OR MH "Narratives" OR MH "Life Experiences" OR MH "Work Experiences" OR MH "Patient Attitudes" OR MH "Attitude to Disability" OR MH "Employee Attitudes" OR MH "Thematic Analysis" OR TI qualitative OR AB qualitative OR TI interview* OR AB interview* OR TI "mixed method" OR AB "mixed method" OR TI "mixed methods" OR AB "mixed methods" | 633,861 |
| S10 | S3 AND S8 AND S9 | 1,082 |
| S11 | EM 19000101-20191126 | 5,965,552 |
| S12 | S10 AND S11 (original) | 737 |
| S13 | S3 AND S8 AND S11 (revised) | 3,881 |

**ICU sustainability – original Ovid Medline search strategy**

1 exp Intensive Care Units/ 114277

2 exp Critical Care/ 69312

3 'critical care'.ti,ab,kf,jw. 154689

4 'intensive care'.ti,ab,kf,jw. 237318

5 'coronary care'.ti,ab,kf,jw. 5300

6 (ICU or picu or (nicu not catalyst)).ti,ab,kf. 119872

7 or/1-6 427549

8 "Conservation of Natural Resources"/ 52869

9 Climate Change/ 32198

10 Global Warming/ 5037

11 Carbon Footprint/ 1487

12 Waste Management/ 10219

13 medical waste/ or medical waste disposal/ 3683

14 Disposable Equipment/ 5406

15 Equipment Reuse/ 3298

16 Recycling/ 7445

17 (environment* adj1 (sustain* or footprint)).ti,ab,kf. 9907

18 (environment* adj friendly).ti,ab,kf. 28951

19 (ecologic* adj1 (sustain* or friendly or footprint)).ti,ab,kf. 2249

20 (eco adj1 (sustain* or friendly or footprint)).ti,ab,kf. 18201

21 ecofriendly.ti,ab,kf. 2699

22 'climate change'.ti,ab,kf. 76002

23 'climate emergency'.ti,ab,kf. 200

24 'global warming'.ti,ab,kf. 15955

25 'carbon footprint'.ti,ab,kf. 3410

26 'net zero'.ti,ab,kf. 1167

27 'zero carbon'.ti,ab,kf. 433

28 'planetary health'.ti,ab,kf. 1524

29 or/8-28 225413

30 7 and 29 423

31 ((green or greener or greening) and (icu or picu or (nicu not catalyst) or 'intensive care' or 'critical care' or 'coronary care')).ti. 19

32 30 or 31 437

33 limit 32 to dt=19500101-20241004 412

**ICU sustainability – revised Ovid Medline search strategy**

1 exp Intensive Care Units/ 114277

2 exp Critical Care/ 69312

3 'critical care'.ti,ab,kf,jw. 154689

4 'intensive care'.ti,ab,kf,jw. 237318

5 'coronary care'.ti,ab,kf,jw. 5300

6 (ICU or picu or (nicu not catalyst)).ti,ab,kf. 119872

7 or/1-6 427549

8 "Conservation of Natural Resources"/ 52869

9 Climate Change/ 32198

10 Global Warming/ 5037

11 Carbon Footprint/ 1487

12 Waste Management/ 10219

13 medical waste/ or medical waste disposal/ 3683

14 Disposable Equipment/ 5406

15 Equipment Reuse/ 3298

16 Recycling/ 7445

17 environment*.ti,ab,kf. 1532348

18 ecologic*.ti,ab,kf. 186625

19 eco.ti,ab,kf. 32218

20 ecofriendly.ti,ab,kf. 2699

21 'climate change'.ti,ab,kf. 76002

22 'climate emergency'.ti,ab,kf. 200

23 'global warming'.ti,ab,kf. 15955

24 'carbon footprint'.ti,ab,kf. 3410

25 'waste management'.ti,ab,kf. 10467

26 'medical waste'.ti,ab,kf. 1370

27 disposable.ti,ab,kf. 17401

28 reuse.ti,ab,kf. 19017

29 recycling.ti,ab,kf. 52144

30 'net zero'.ti,ab,kf. 1167

31 'zero carbon'.ti,ab,kf. 433

32 'planetary health'.ti,ab,kf. 1524

33 (green or greener or greening).ti. 59743

34 or/8-33 1875784

35 7 and 34 14149

36 limit 35 to dt=19300101-20241004 13812

**Reproductive coercion – original & revised Ovid Medline search strategy**

1  Disabled Persons/ (49866)
2  (disabled or disabilit* or handicap*).tw,kf. (316170)
3  Developmental Disabilities/ (23263)
4  (development* adj2 (disorder* or impair*)).tw,kf. (28194)
5  Neurodevelopmental Disorders/ (5714)
6  (neurodevelopment* adj2 (disorder* or impair*)).tw,kf. (24435)
7  Attention Deficit Disorder with Hyperactivity/ (37015)
8  (attention deficit disorder* or attention deficit hyperactivity disorder* or adhd).tw,kf. (45859)
9  Autism Spectrum Disorder/ (23343)
10  (autis* or asperger* or neurodivers* or neuro divers*).tw,kf. (77175)
11  exp Learning Disorders/ (24525)
12  (learning disorder* or dyslexi*).tw,kf. (9021)
13  exp Intellectual Disability/ or Persons with Mental Disabilities/ (111634)
14  (intellectual* adj1 (disorder* or impair* or delay* or retard*)).tw,kf. (3963)
15  (mental* adj1 (disorder* or impair* or delay* or retard*)).tw,kf. (103529)
16  (cognitive* adj1 (disorder* or impair* or delay*)).tw,kf. (120117)
17  (down* syndrome or fragile x syndrome or klinefelter syndrome or williams syndrome or Prader Willi syndrome).tw,kf. (39492)
18  Fetal Alcohol Spectrum Disorders/ (4837)
19  (f?etal alcohol or fasd).tw,kf. (5555)
20  exp Blindness/ or Visually Impaired Persons/ (29289)
21  (blindness or (blind* adj2 (individual* or people or person* or woman* or women))).tw,kf. (41478)
22  (visual* adj2 (disorder* or impair*)).tw,kf. (24766)
23  (vision adj2 (disorder* or impair*)).tw,kf. (7441)
24  exp Deafness/ or Persons With Hearing Impairments/ (33918)
25  (deaf* or 'hard of hearing').tw,kf. (44167)
26  (hearing adj2 (disorder* or impair*)).tw,kf. (22876)
27  Mental Disorders/ or exp Dissociative Disorders/ or exp "Bipolar and Related Disorders"/ or Depressive Disorder, Major/ or exp Personality Disorders/ or exp "Schizophrenia Spectrum and Other Psychotic Disorders"/ or Mentally Ill Persons/ (447907)
28  (mental* adj1 (ill* or disorder* or condition* or problem*)).tw,kf. (116241)
29  (psychiatric adj1 (ill* or disorder* or condition* or problem*)).tw,kf. (77092)
30  (dissociative disorder* or schizophrenia or bipolar or borderline or psychotic or psychos?s).tw,kf. (310917)
31  exp Chronic Disease/ (657291)
32  (chronic* adj (disease* or ill or illn* or condition*)).tw,kf. (147367)
33  (episodic adj (disease* or ill or illn* or condition*)).tw,kf. (235)
34  exp Arthritis/ or arthritis.tw,kf. (385621)
35  Cerebral Palsy/ or cerebral palsy.tw,kf. (34401)
36  Cystic Fibrosis/ or cystic fibrosis.tw,kf. (59691)
37  Fatigue Syndrome, Chronic/ or chronic fatigue.tw,kf. (10114)
38  Crohn Disease/ or crohn*.tw,kf. (71154)
39  exp Epilepsy/ or epilepsy.tw,kf. (180789)
40  Fibromyalgia/ or fibromyalgia.tw,kf. (15361)
41  exp HIV/ or exp HIV Infections/ or hiv.tw,kf. (459789)
42  exp Lupus Erythematosus, Systemic/ or lupus.tw,kf. (107014)
43  Amyotrophic Lateral Sclerosis/ or amyotrophic lateral sclerosis.tw,kf. or lou gehrig*.tw,kf. (37410)
44  exp Multiple Sclerosis/ or multiple sclerosis.tw,kf. (107352)
45  exp Parkinson Disease/ or parkinson*.tw,kf. (168858)
46  exp Colitis, Ulcerative/ or ulcerative colitis.tw,kf. (64611)
47  exp Movement Disorders/ (169189)
48  (mobility adj2 (issue* or problem* or impair*)).tw,kf. (4931)
49  Amputees/ or amputee*.tw,kf. (8597)
50  exp Spinal Cord Injuries/ or spinal cord injur*.tw,kf. (74812)
51  exp Paraplegia/ or exp Quadriplegia/ or exp Paresis/ (29527)
52  (paraplegi* or quadriplegi*).tw,kf. (24206)
53  Wheelchairs/ (5706)
54  wheelchair*.tw,kf. (9437)
55  or/1-54 (3773248)
56  Coercion/ or Involuntary Treatment/ (5391)
57  Reproduction/ or Reproductive Health/ or Abortion, Induced/ or Pregnancy/ or Pregnancy, Unplanned/ or Reproductive Health Services/ or Maternal Health Services/ or Family Planning Services/ or Delivery, Obstetric/ or exp Contraception/ or exp Contraceptive Agents/ (1191799)
58  56 and 57 (931)
59  Reproductive Rights/ (1265)
60  Sterilization, Involuntary/ (710)
61  Violence/ and Delivery, Obstetric/ (94)
62  (reproductive adj (rights or justice)).tw,kf. (1867)
63  (reproductive adj1 (coerc* or violence)).tw,kf. (257)
64  (obstetric* adj1 (coerc* or violence)).tw,kf. (290)
65  (forced adj1 (sterili?ation or birth control or contraception or contraceptive* or abortion or pregnancy)).tw,kf. (153)
66  (involuntar* adj1 (sterili?ation or birth control or contraception or contraceptive* or abortion or pregnancy)).tw,kf. (139)
67  (sabotag* adj1 (birth control or contraception or contraceptive*)).tw,kf. (44)
68  or/58-67 (4783)
69  55 and 68 (964)
70  limit 69 to dt=19650101-20230320 (904) (original)
71  Disabled Persons/ (49866)
72  (disabled or disabilit* or handicap*).tw,kf. (316170)
73  Developmental Disabilities/ (23263)
74  (development* and (disorder* or impair*)).tw,kf. (374427)
75  Neurodevelopmental Disorders/ (5714)
76  (neurodevelopment* and (disorder* or impair*)).tw,kf. (39396)
77  Attention Deficit Disorder with Hyperactivity/ (37015)
78  (attention deficit disorder* or attention deficit hyperactivity disorder* or adhd).tw,kf. (45859)
79  Autism Spectrum Disorder/ (23343)
80  (autis* or asperger* or neurodivers* or neuro divers*).tw,kf. (77175)
81  exp Learning Disorders/ (24525)
82  (learning disorder* or dyslexi*).tw,kf. (9021)
83  exp Intellectual Disability/ or Persons with Mental Disabilities/ (111634)
84  (intellectual* and (disorder* or impair* or delay* or retard*)).tw,kf. (26504)
85  (mental* adj1 (disorder* or impair* or delay* or retard*)).tw,kf. (103529)
86  (cognitive* and (disorder* or impair* or delay*)).tw,kf. (259054)
87  (down* syndrome or fragile x syndrome or klinefelter syndrome or williams syndrome or Prader Willi syndrome).tw,kf. (39492)
88  Fetal Alcohol Spectrum Disorders/ (4837)
89  (f?etal alcohol or fasd).tw,kf. (5555)
90  exp Blindness/ or Visually Impaired Persons/ (29289)
91  (blindness or (blind* and (individual* or people or person* or woman* or women))).tw,kf. (112794)
92  (visual* and (disorder* or impair*)).tw,kf. (96545)
93  (vision and (disorder* or impair*)).tw,kf. (25985)
94  exp Deafness/ or Persons With Hearing Impairments/ (33918)
95  (deaf* or 'hard of hearing').tw,kf. (44167)
96  (hearing and (disorder* or impair*)).tw,kf. (38065)
97  Mental Disorders/ or exp Dissociative Disorders/ or exp "Bipolar and Related Disorders"/ or Depressive Disorder, Major/ or exp Personality Disorders/ or exp "Schizophrenia Spectrum and Other Psychotic Disorders"/ or Mentally Ill Persons/ (447907)
98  (mental* and (ill* or disorder* or condition* or problem*)).tw,kf. (292330)
99  (psychiatric and (ill* or disorder* or condition* or problem*)).tw,kf. (165058)
100  (dissociative disorder* or schizophrenia or bipolar or borderline or psychotic or psychos?s).tw,kf. (310917)
101  exp Chronic Disease/ (657291)
102  (chronic* and (disease* or ill or illn* or condition*)).tw,kf. (850531)
103  (episodic and (disease* or ill or illn* or condition*)).tw,kf. (12959)
104  exp Arthritis/ or arthritis.tw,kf. (385621)
105  Cerebral Palsy/ or cerebral palsy.tw,kf. (34401)
106  Cystic Fibrosis/ or cystic fibrosis.tw,kf. (59691)
107  Fatigue Syndrome, Chronic/ or chronic fatigue.tw,kf. (10114)
108  Crohn Disease/ or crohn*.tw,kf. (71154)
109  exp Epilepsy/ or epilepsy.tw,kf. (180789)
110  Fibromyalgia/ or fibromyalgia.tw,kf. (15361)
111  exp HIV/ or exp HIV Infections/ or hiv.tw,kf. (459789)
112  exp Lupus Erythematosus, Systemic/ or lupus.tw,kf. (107014)
113  Amyotrophic Lateral Sclerosis/ or amyotrophic lateral sclerosis.tw,kf. or lou gehrig*.tw,kf. (37410)
114  exp Multiple Sclerosis/ or multiple sclerosis.tw,kf. (107352)
115  exp Parkinson Disease/ or parkinson*.tw,kf. (168858)
116  exp Colitis, Ulcerative/ or ulcerative colitis.tw,kf. (64611)
117  exp Movement Disorders/ (169189)
118  (mobility and (issue* or problem* or impair*)).tw,kf. (25582)
119  Amputees/ or amputee*.tw,kf. (8597)
120  exp Spinal Cord Injuries/ or spinal cord injur*.tw,kf. (74812)
121  exp Paraplegia/ or exp Quadriplegia/ or exp Paresis/ (29527)
122  (paraplegi* or quadriplegi*).tw,kf. (24206)
123  Wheelchairs/ (5706)
124  wheelchair*.tw,kf. (9437)
125  or/71-124 (4637771)
126  Coercion/ or Involuntary Treatment/ (5391)
127  Reproduction/ or Reproductive Health/ or Abortion, Induced/ or Pregnancy/ or Pregnancy, Unplanned/ or Reproductive Health Services/ or Maternal Health Services/ or Family Planning Services/ or Delivery, Obstetric/ or exp Contraception/ or exp Contraceptive Agents/ (1191799)
128  126 and 127 (931)
129  Reproductive Rights/ (1265)
130  Sterilization, Involuntary/ (710)
131  Violence/ and Delivery, Obstetric/ (94)
132  (reproductive and (rights or justice)).tw,kf. (4698)
133  (reproductive and (coerc* or violence)).tw,kf. (3058)
134  (obstetric* and (coerc* or violence)).tw,kf. (1136)
135  (forced and (sterili?ation or birth control or contraception or contraceptive* or abortion or pregnancy)).tw,kf. (1536)
136  (involuntar* and (sterili?ation or birth control or contraception or contraceptive* or abortion or pregnancy)).tw,kf. (569)
137  (sabotag* and (birth control or contraception or contraceptive*)).tw,kf. (48)
138  or/128-137 (11622)
139  125 and 138 (2772)
140  limit 139 to dt=19650101-20230320 (2448) (revised)

**ED flow training – original and revised Ovid Medline search strategy**

1  exp Emergency Service, Hospital/ (106480)
2  Emergency Medical Services/ (50925)
3  Emergency Services, Psychiatric/ (2595)
4  (emergency adj2 (department? or unit? or room?)).ti,ab. (170168)
5  (trauma adj2 (centre? or center? or department? or unit?)).ti,ab. (26878)
6  (triage adj2 (centre? or center? or department? or unit?)).ti,ab. (940)
7  ("accident and emergency" or "accident & emergency" or 'emergency service?').ti,ab. (13035)
8  or/1-7 (271526)
9  exp Emergency Medicine/ (16227)
10  Physicians/ (106469)
11  Physician Assistants/ (6627)
12  Emergency Nursing/ (7635)
13  exp Nurses/ (102231)
14  Nursing Assistants/ (4484)
15  exp Medical Staff/ (29344)
16  Health Personnel/ (71628)
17  Allied Health Personnel/ (13490)
18  Emergency Medical Technicians/ (6187)
19  ('emergency medical technician*' or paramedic?).ti,ab. (8164)
20  (emergency adj5 (nurse? or physician? or resident? or clinician? or provider? or technician? or staff or personnel)).ti,ab. (36619)
21  (ed adj (nurse? or physician? or resident? or clinician? or provider? or technician? or staff or personnel)).ti,ab. (4631)
22  or/9-21 (375112)
23  exp Professional Competence/ (135836)
24  (skill* or competenc* or knowledge).ti,ab. (1329629)
25  Education, Medical, Continuing/ (25740)
26  Education, Nursing, Continuing/ (23843)
27  ed.fs. (312766)
28  inservice training/ (20827)
29  staff development/ (10079)
30  exp Simulation Training/ (13060)
31  (education* or train* or instruct* or learn*).ti,ab. (2029865)
32  (workshop* or 'work shop*' or webinar?).ti,ab. (54228)
33  exp Communication/ (385564)
34  communication.ti,ab. (341461)
35  cooperative behavior/ (47480)
36  helping behavior/ (3336)
37  exp Patient Care Team/ (74725)
38  (teamwork or 'team work').ti,ab. (16376)
39  exp Interprofessional Relations/ (73597)
40  ('interprofessional relation*' or 'inter professional relation*').ti,ab. (570)
41  ('intraprofessional relations*' or 'intra professional relations*').ti,ab. (36)
42  "Attitude of Health Personnel"/ (137213)
43  attitude?.ti,ab. (209984)
44  leadership/ (50098)
45  leadership.ti,ab. (56414)
46  assertiveness/ (1787)
47  assertiveness.ti,ab. (2131)
48  Awareness/ (22748)
49  'situation* awareness'.ti,ab. (2356)
50  anticipating.ti,ab. (7270)
51  Decision Making/ (108746)
52  (decisionmaking or 'decision making').ti,ab. (221848)
53  time management/ (3035)
54  ('time management' or 'task management' or 'workload management').ti,ab. (3192)
55  ('task distribution' or 'distribut* tasks').ti,ab. (167)
56  Guideline Adherence/ (36214)
57  adherence.ti,ab. (176993)
58  ('mutual standard maintenance' or 'maintaining standard?').ti,ab. (211)
59  (provid* adj (feedback or 'feed back')).ti,ab. (6761)
60  "Task Performance and Analysis"/ (33474)
61  workflow/ (10185)
62  Quality Improvement/ (35511)
63  total quality management/ (12805)
64  'quality improvement'.ti,ab. (54629)
65  lean.ti,ab. (48873)
66  'process improvement'.ti,ab. (2400)
67  'design thinking'.ti,ab. (650)
68  streaming.ti,ab. (6791)
69  or/23-68 (4374200)
70  Waiting Lists/ (14544)
71  wait*.ti,ab. (79707)
72  Time-to-Treatment/ (10904)
73  ((time or timeliness) adj2 treatment).ti,ab. (39539)
74  ((decreas* or reduce* or reduction) adj2 time).ti,ab. (85798)
75  'response time?'.ti,ab. (27683)
76  "Length of Stay"/ (108793)
77  ('length of stay' or los).ti,ab. (145608)
78  efficiency/ (16065)
79  efficiency, organizational/ (22673)
80  efficien*.ti,ab. (1448414)
81  (throughput or 'through put').ti,ab. (158196)
82  (turnaround or 'turn around').ti,ab. (9850)
83  flow.ti,ab. (881286)
84  or/70-83 (2845432)
85  8 and 22 and 69 and 84 (3051)
86  limit 85 to dt=19500101-20220118 (2565) (original)
87  8 and 22 and 84 (5645)
88  limit 87 to dt=19500101-20220118 (4753) (revised)

**AI ethics – original Scopus search strategy**

( ( TITLE-ABS ( teach* OR instruct* OR tutor* OR educat* OR pedagog* OR paedagog* OR curriculum* OR course OR courses OR coursework OR workshop* OR "co op" OR "information literacy" OR "computer literacy" OR universit* OR college* OR postsecondary OR "post secondary" OR "higher education" OR undergraduate* ) OR SRCTITLE ( teach* OR educat* OR pedagog* OR paedagog* OR curriculum* OR universit* OR college* OR postsecondary OR "post secondary" OR "higher education" ) ) AND ( TITLE-ABS ( ethic* OR moral* OR "human right*" OR "civil right*" OR harm OR harms OR privacy OR confidentiality OR consent OR "right to erasure" OR "right to be forgotten" OR "right to information" OR "data protection" OR equality OR equity OR diversity OR inclusivity OR inclusiveness OR fairness OR bias OR biases OR biased OR discrimination OR prejudic* OR racist* OR racism* OR sexist* OR sexism OR homophob* OR "homo phob*" OR transphob* OR "trans phob*" OR harassment* OR "cultural competenc*" OR paternalism OR decoloniz* OR "de coloniz*" OR decolonis* OR "de colonis*" OR accountability OR verifiability OR replicability OR transparent OR transparency OR explainability OR trustworthy* OR "environmental responsibility" OR "environmentally responsible" OR security OR "open source" OR professionalism OR "professional responsibility" OR integrity OR "human control" ) ) AND ( TITLE-ABS ( "Artificial intelligence" OR "Machine learning" OR "deep learning" OR "artificial neural network*" OR "large language model*" OR alexnet OR "generative adversarial network*" OR biggan OR stylegan OR resnets OR alphago OR "Generative Pretrained Transformer" OR gpt OR chatgpt OR "Bidirectional Encoder Representations from Transformers" OR bert OR "self supervised learning" OR alphafold OR "Dall e" OR "github copilot" OR "Stable diffusion" OR llama OR alpaca OR bard OR "palm 2" ) OR TITLE ( ai OR computer* OR computing OR algorithm* OR "data mining" ) ) ) AND ( LOAD-DATE < 20240626 )

**AI ethics – revised Scopus search strategy**

( ( TITLE-ABS ( universit* OR college* OR postsecondary OR "post secondary" OR "higher education" OR undergraduate* ) OR ( TITLE-ABS (engineering OR "computer science" OR nursing OR medical) W/5 (student* OR course* OR curricul*)) OR SRCTITLE ( teach* OR educat* OR pedagog* OR paedagog* OR curriculum* OR universit* OR college* OR postsecondary OR "post secondary" OR "higher education" ) ) AND ( TITLE-ABS ( ethic* OR moral* OR "human right*" OR "civil right*" OR harm OR harms OR privacy OR confidentiality OR consent OR "right to erasure" OR "right to be forgotten" OR "right to information" OR "data protection" OR equality OR equity OR diversity OR inclusivity OR inclusiveness OR fairness OR bias OR biases OR biased OR discrimination OR prejudic* OR racist* OR racism* OR sexist* OR sexism OR homophob* OR "homo phob*" OR transphob* OR "trans phob*" OR harassment* OR "cultural competenc*" OR paternalism OR decoloniz* OR "de coloniz*" OR decolonis* OR "de colonis*" OR accountability OR verifiability OR replicability OR transparent OR transparency OR explainability OR trustworthy* OR "environmental responsibility" OR "environmentally responsible" OR security OR "open source" OR professionalism OR "professional responsibility" OR integrity OR "human control" ) ) AND ( TITLE-ABS ( "Artificial intelligence" OR "Machine learning" OR "deep learning" OR "artificial neural network*" OR "large language model*" OR alexnet OR "generative adversarial network*" OR biggan OR stylegan OR resnets OR alphago OR "Generative Pretrained Transformer" OR gpt OR chatgpt OR "Bidirectional Encoder Representations from Transformers" OR bert OR "self supervised learning" OR alphafold OR "Dall e" OR "github copilot" OR "Stable diffusion" OR llama OR alpaca OR bard OR "palm 2" ) OR TITLE ( ai OR computer* OR computing OR algorithm* OR "data mining" ) ) ) AND ( LOAD-DATE < 20240626 )
