# Supplementary material for: Comparing the performance of narrow vs. broad search strategies when using machine learning-based software for title/abstract screening
Source: J Med Libr Assoc. 2026 Apr 13;114(2):105–15. doi: 10.5195/jmla.2026.2286 (PMC13075572; doi:10.5195/jmla.2026.2286)
Supplement: Supplementary file 2 — Appendix B: Figures, Etc. [file jmla-114-2-105-s02.docx]

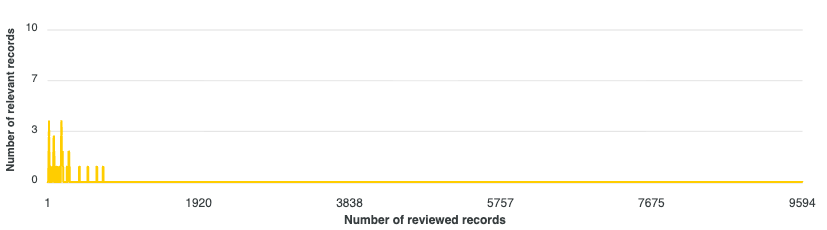


**Figure 1 – Simulation progress graph for the AI Ethics narrow search strategy (run #1).**


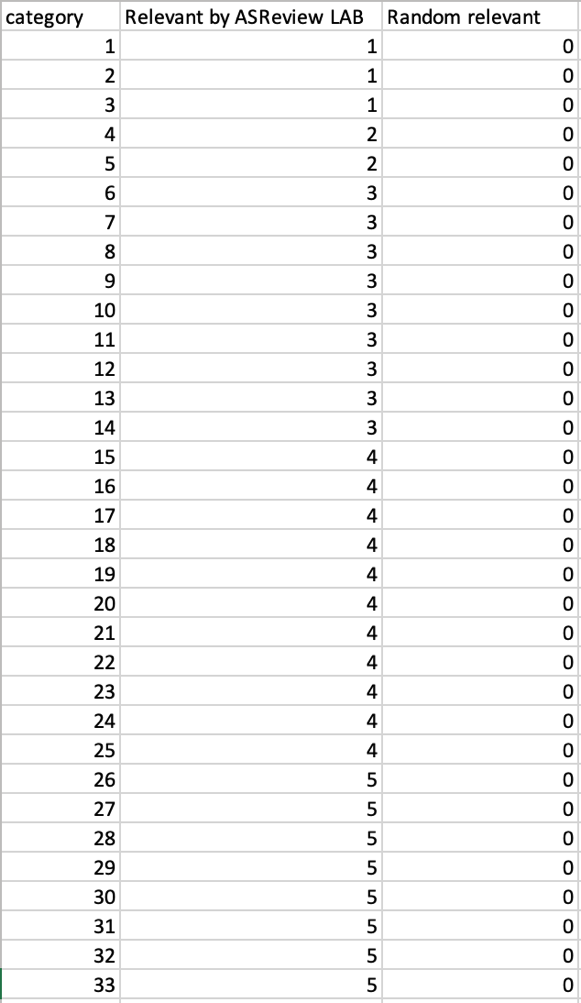


**Figure 2 – Partial simulation result output for the AI Ethics narrow search strategy (run #1).** The column “category” shows the number of articles reviewed by the active learning algorithm. “Relevant by ASReview LAB” represents the total number of included articles identified when using active learning re-prioritization, while “Random relevant” indicates the total number of included articles found without re-prioritization.
